# Hempseed Water-Soluble Protein Fraction and Its Hydrolysate Display Different Biological Features

**DOI:** 10.3390/life15020225

**Published:** 2025-02-04

**Authors:** Annalisa Givonetti, Stelvio Tonello, Chiara Cattaneo, Davide D’Onghia, Nicole Vercellino, Pier Paolo Sainaghi, Donato Colangelo, Maria Cavaletto

**Affiliations:** 1 Dipartimento per lo Sviluppo Sostenibile e la Transizione Ecologica, Università del Piemonte Orientale, Piazza S. Eusebio 5, 13100 Vercelli, Italy; maria.cavaletto@uniupo.it; 2 Dipartimento di Medicina Traslazionale, Università del Piemonte Orientale, Via Solaroli 17, 28100 Novara, Italy; stelvio.tonello@med.uniupo.it (S.T.); davide.donghia@uniupo.it (D.D.); nicole.vercellino@uniupo.it (N.V.); pierpaolo.sainaghi@med.uniupo.it (P.P.S.); 3 Università del Piemonte Orientale, Piazza S. Eusebio 5, 13100 Vercelli, Italy; chiara.cattaneo@uniupo.it; 4 Dipartimento di Scienze della Salute, Farmacologia, Scuola di Medicina, Università del Piemonte Orientale, Via Solaroli 17, 28100 Novara, Italy

**Keywords:** *Cannabis sativa*, hempseed, water-soluble protein, antioxidant, SDS-PAGE, MALDI-TOF, hempseeds hydrolysates, peptidomics, in vitro digestion, autoimmune disease, IBD

## Abstract

Hempseeds, from the *Cannabis sativa* plant, and its derivates are a versatile food option for various dietary preferences. Due to their aminoacidic profile, researchers have studied the presence of bioactive peptides in hempseed proteins. In this study, the water-soluble fraction of hempseed protein was extracted, and the derived peptides were analyzed. The investigation focused on their biological function, particularly their antioxidant activity. Several biological functions have arisen, such as angiotensin-converting enzyme inhibition activity, dipeptidyl-peptidase IV, dipeptidyl-peptidase III inhibition, and ubiquitin-mediated proteolysis activation. The hydrolysates show greater 2,2-azinobis-[3-ethylbenzothiazoline-6-sulphonic acid (ABTS) radical scavenging activity compared to the proteins (97.95 ± 4.48 versus 81.04 ± 10.63). Furthermore, the impact of these proteins and peptides on the U937 cell line was evaluated to assess cell viability and their potential role in modulating inflammation associated with gastrointestinal autoimmune diseases. Protein treatment resulted in a significant reduction in cell viability, as opposed to hydrolysates, which did not affect it.

## 1. Introduction

Hempseeds, from the *Cannabis sativa* L. plant, are a (re)emerging food. Not only can they be consumed whole (hulled seeds) or dehulled (hemp heart), but they are also processed into oil, flour, or protein powder, offering a precious range of dietary options [1]. The consumption of hemp seeds as a source of food has been reported throughout history [2] until the first half of the 21st century when the cultivation of hemp was banned due to the presence of delta-9-tetrahydrocannabinol (THC), a compound with psychoactive and toxicant properties [3]. Recently, some Western countries have allowed the cultivation of specific varieties of *Cannabis sativa* with a low content of THC (<0.2% in Europe), owing to the diverse application of the hemp plant [4].

The nutritional composition of hempseeds depends on factors such as the variety under analysis and the geographical location where they have been grown [5]. However, hempseeds are considered complete from a nutritional point of view: they contain a good amount of lipids (25–35%), proteins (20–25%), carbohydrates (20–30%), insoluble fibers (10–15%), vitamins, and minerals [6]. The oil extracted from these seeds is notably abundant in polyunsaturated fatty acids, mainly linoleic acid and a-linolenic acid, with an omega-6/omega-3 ratio of 3:1, considered optimal for human nutrition [7]. Although other proteins have been identified, the main seed storage proteins are albumin and edestin. What distinguishes this food source from other plant-based foods is the presence of all the essential amino acids required by humans and high levels of arginine, which plays a dual role in immune regulation by modulating the inflammatory response [8], and glutamine, which supports intestinal epithelium integrity and immune cell function [9,10,11]. In addition, hemp seed proteins are less allergenic, making it a safer option for people sensitive or prone to food-related immune reactions [12] and easier to digest than soy protein [13]. The hydrolysis of hempseed proteins results in hydrolysates that exert different biological activities, such as antioxidant [14,15], antihypertensive [16,17], and anti-inflammatory properties [17,18]. These peptides may influence key pathways involved in autoimmune diseases, such as reducing pro-inflammatory cytokines and oxidative stress. These are critical in the pathogenesis of autoimmune disorders such as inflammatory bowel diseases, rheumatoid arthritis, and multiple sclerosis.

The U937 is a human myeloid leukemia cell line with a pro-monocytic phenotype, which is widely used in biomedical research. These cells are extensively used to study the biology of monocytes and macrophages, as they can be differentiated into macrophage-like cells under specific growth conditions. This characteristic makes U937 cells an excellent in vitro model for investigating immune responses, including inflammatory processes relevant to autoimmune diseases [19,20]. U937 cells are employed in autoimmune conditions to elucidate the mechanisms of immune dysregulation and inflammatory signaling pathways. This cell line represents a model to study macrophage activation, cytokine secretion, and the interaction between immune cells and environmental stimuli. For instance, they are employed to simulate the behavior of tissue-resident macrophages in diseases such as systemic lupus erythematosus (SLE), rheumatoid arthritis (RA), and inflammatory bowel diseases (IBDs), including Crohn’s disease and ulcerative colitis [19]. Furthermore, the U937 cell line has been used to evaluate the immunomodulatory effects of various bioactive compounds, including protein hydrolysates derived from dietary sources like hemp seeds. These studies are essential to understanding how bioactive peptides can modulate macrophage activity and contribute to mitigating inflammation. By serving as a proxy for immune cells in the gut-associated immune system, U937 cells offer insights into the interactions between dietary components and the immune system in autoimmune disorders [21,22,23].

Autoimmune diseases affecting the gastrointestinal (GI) tract involve aberrant immune activation against host tissues, leading to chronic inflammation and tissue damage. Prominent examples include IBDs, such as Crohn’s disease and ulcerative colitis, and celiac disease, which involves an immune response to dietary gluten [24].

In IBDs, the immune system fails to maintain tolerance to the intestinal microbiota, resulting in excessive activation of immunological pathways. This leads to the recruitment and activation of macrophages, dendritic cells, and T lymphocytes, which drive the release of pro-inflammatory cytokines such as TNF-α, IL-6, and IL-1β. These inflammatory mediators contribute to the disruption of the epithelial barrier and the perpetuation of chronic inflammation [25]. The U937 cell line has been instrumental in mimicking these immune responses, offering a controlled system to study cytokine production and macrophage polarization under inflammatory conditions [19,20].

In celiac disease, ingesting gluten triggers an inappropriate immune response characterized by tissue transglutaminase activation and gliadin peptide presentation by HLA-DQ2/DQ8 molecules. This response activates CD4+ T cells, leading to the production of autoantibodies and the destruction of intestinal villi [24]. Investigating how dietary proteins or peptides influence immune activation in this context is crucial, especially to evaluate the potential of bioactive peptides in modulating inflammatory responses [23].

Although numerous studies have already described the biological activity of hempseed proteins and hydrolysates, a knowledge gap exists about proteins and peptides obtained through methods simulating human digestion. Moreover, most studies focus on the effects of isolated peptides or specific fractions obtained after enzymatic digestion [26,27,28,29] without considering the entire spectrum of peptides generated during digestion. Based on the seed-storage protein classification formulated by Osborne [30], water-soluble proteins can be extracted, representing a physiologically relevant fraction as they are solubilized in a solution closely resembling saliva. By focusing on this fraction, we aim to investigate whether its hydrolysates show enhanced biological activity compared to the intact protein fraction. Simulating gastric digestion using pepsin [31] allows us to mimic the initial digestive process and evaluate how digestion modifies the bioactivity of these proteins. This approach will help bridge the gap between in vitro enzymatic studies and in vivo relevance, highlighting the potential therapeutic benefits of hemp-derived peptides in inflammatory conditions.

To further correlate these results with a potential role in immunomodulation upon the arrival of hempseed hydrolysates in the gut, we selected the human monocyte cell line U937 as an experimental model to investigate macrophage function. This approach enables us to assess the potential therapeutic benefits of hemp-derived peptides in modulating autoimmune diseases with GI involvement [21,22,23].

## 2. Materials and Methods

### 2.1. Protein Extraction

Partially de-oiled hempseed flour [Koro commercial brand, protein 49%, carbohydrate 4.2%, fat 11%] was used to conduct the experiments. The flour was purchased from the producer’s website (koro-shop.it).

Water-soluble protein (WsP) extraction was carried out as indicated in [32] with some modifications. Briefly, the flour was subjected to further delipidation with n-hexane (VWR CHEMICALS, ≥97%, HiPerSolv CHROMANORM® for HPLC) in a 1:6 ratio (*w*/*v*). The tubes were incubated overnight at room temperature, keeping the samples stirred on an orbital shaker at 250 rpm (Multi-functional Orbital Shaker PSU-20i, bioSan, Riga, Latavia). The pellet was dried after transferring the lipid part (Concentrator plus, Eppendorf). The WsP fraction was obtained by adding to the pellet ultrapure water in a 1:10 ratio (*w*/*v*). The mixture was mixed by vortexing for 5 min and then left to stir at 4 °C for 55 min. After a centrifuge step (12,000× *g*, 10 min, 4 °C), the supernatant was transferred and stocked at 4 °C. The extraction was repeated twice, and the supernatants were pooled. The WsP fraction was concentrated (Concentrator plus, Eppendorf) and then subjected to a precipitation step with ethanol 60%. The precipitation was conducted overnight at −20 °C. The mixture was centrifuged (18,000× *g*, 15 min, 4 °C), and the supernatant was discharged. The ethanol residue was evaporated using Concentrator plus. The pellet was resuspended in water using half the original volume. The protein content was estimated using a Bradford assay [33] with bovine serum albumin (BSA) as the protein standard. The extraction was conducted in duplicate.

### 2.2. Hydrolysate Production

A part of WsP was subjected to hydrolysis with pepsin (Pepsin from porcine gastric mucosa, (SIGMA ALDRICH, Merck Life Science S.r.l., Milano, Italy), as indicated in [29], with some modifications. Briefly, the pH of the sample was adjusted to 2 by adding 1 M HCl. The enzyme solution was prepared (4 mg/mL in 30 mM NaCl) and added to the WsP in a 1:50 enzyme/protein ratio (*w*/*w*). The mixture was incubated at 37 °C for 2 h. The hydrolysis was stopped, changing the pH to 7.8 with 1 M NaOH. The hydrolysate production was performed in duplicate.

### 2.3. Powder Production

The protein fraction and the hydrolysate samples obtained with the extraction and production method described above were lyophilized (CoolSafe Freeze Dryer, Labo-Gene, Bjarkesvej Denmark) to obtain a powder ready to be resuspended in the most suitable buffer.

### 2.4. SDS-PAGE

Proteins and hydrolysates powder were resuspended in ultrapure water, and the solution was filtered with a 0.22 µm filter. The samples were analyzed using SDS-PAGE (sodium dodecyl sulfate–polyacrylamide gel electrophoresis): 10 µg of proteins and an equal volume of hydrolysates were mixed with a Laemmli buffer [34] (2% *w*/*v* SDS, 10% glycerol, 5% 2-mercaptoethanol, 62 mM Tris-HCl pH 6.8) and loaded onto 8.6 × 6.7 cm vertical 4–15% precast polyacrylamide gel. The protein standard (Precision Plus ProteinTM Dual Xtra Standards, Biorad, Segrate-Milano, Italy) was also loaded to estimate the molecular weight of proteins and hydrolysates. The SDS-PAGE was performed at 15 mA for 30 min and 30 mA with Mini Protean System (BioRad, Segrate-Milano, Italy). The run buffer was 25 mM Tris-HCl, 200 mM glycine, 0.1% *w*/*v* SDS. The bands were stained with a Colloidal Coomassie brilliant blue G250 solution, and the gel image was obtained using a GS-900 densitometer (Biorad, Segrate-Milano, Italy).

### 2.5. MALDI-TOF

A MALDI-TOF analysis was conducted on both proteins and hydrolysates, combining 1.5 µL of the samples with 10 µL of a matrix solution (10 mg/mL sinapinic acid dissolved in acetonitrile: 0.1% TFA, 30:70% *v*/*v*-TA30). A total of 1.0 µL of each mix was spotted on a ground steel MTP 384 target plate (Bruker Daltonics GmbH, Bremen, Germany) in triplicate and dried at room temperature overnight. MALDI-TOF spectra were acquired via an Ultraflextreme mass spectrometer (Bruker Daltonics GmbH, Bremen, Germany) equipped with a Smart-beam 2 Nd:YAG laser operating at 355 nm, using FlexControl software (version 3.3, Bruker Daltonics GmbH, Bremen, Germany). Protein fractions were analyzed in Linear mode and Positive ion acquisition in a mass-to-charge ratio of 2000–20,000. Instead, hydrolysates were analyzed in reflectron mode and positive ion acquisition in a narrower range of 700–3500 *m*/*z*. The mass spectra obtained are 6000 shots for each sample; the random walk feature with partial sample mode was activated. Protein mass spectra were calibrated through “Protein Calibration Standard I” and hydrolysates using “Peptide Calibration Standard I” (Bruker Daltonics GmbH, Bremen, Germany) in FlexAnalysis software (version 3.3, Bruker Daltonics GmbH, Bremen, Germany).

Information about spectra (*m*/*z* and intensity) obtained through the MALDI-TOF technique was subjected to GeenaR, a web tool for reproducible MALDI-TOF analysis (https://proteomics.hsanmartino.it/geenar/, accessed on 1 December 2024). To analyze the principal components of these spectra, the following parameters were used: trimming from 700 to 3500 (for spectra analyzed with reflectron method) or 2000 to 50,000 (for spectra analyzed with linear method); variance stabilization: square root; smoothing: Savitzky–Golay with half window size of 10; baseline removal: TopHat, with half window size of 75; normalization: total ion current (TIC). Peak alignment was conducted using MAD (median absolute deviation) for noise estimation (half window size: 20, SNR: 2) and LOWESS (local weight scatterplot smoothing) for phase correction (mass tolerance: 0.002). Then, the peaks were selected by applying the strict method for binning and a coverage (minimum frequency of peaks for their selection) of 0.5. A principal component analysis was used on the pre-processed mass spectra [35,36].

### 2.6. Hydrolysates Identification and Biological Activity Estimation

The web-accessible tool PeptideMass (https://web.expasy.org/peptide_mass/, accessed on 4 December 2024) was used to identify the hydrolysates after pepsin digestion. The protein sequences were subjected to the following digestion parameters: enzyme pepsin (pH > 2), 2 as a maximum number of missed cleavages, all cysteines in reduced form; methionines were not oxidized, using monoisotopic masses of the occurring amino acid residues giving peptide masses as [M+H]^+^. The molecular weights of the peptides obtained through in silico digestion were compared with those obtained from the MALDI-TOF analysis of the hydrolysates. Molecular weights in the range ±0.5 Da were deemed equivalent. The obtained peptides were then submitted to the BIOPEP (https://biochemia.uwm.edu.pl/biopep/start_biopep.php, accessed on 10 December 2024) web database to estimate the biological activity of our hydrolysates. Results are reported as a relative abundance of different biological activities. Abundance was calculated as the number of peptides with a given biological function out of the total number of peptides identified for that protein.

### 2.7. Antioxidant Activity Estimation Assay

The radical scavenging activity of the protein and hydrolysate fractions was evaluated using an ABTS (2,2-azinobis-[3-ethylbenzothiazoline-6-sulphonic acid) method adapted for 96-well microplates. The ABTS antioxidant activity was performed as indicated in [35]. Briefly, the ABTS (7 mM) was mixed with potassium persulfate (4.9 mM) in a 1:1 ratio and incubated in a dark place at room temperature overnight. From the reaction of the ABTS and potassium persulfate, the radical cation ABTS (ABTS•+) originated. The solution was filtered (0.22 µm) and diluted with ultrapure water to absorb 0.70 at 734 nm. Proteins and hydrolysates powder were resuspended in ultrapure water in a 1:1 (*w*/*v*) ratio, and the solutions were filtered with a 0.22 µm filter. The antioxidant reaction was carried out by mixing 190 µL of ABTS•+ with 20 µL of the diluted sample: the mixture was incubated in the dark at room temperature for 7 min and then transferred into a 96-well microplate. The absorbance was read at 734 nm with a microplate reader (Spark 10 M, Tecan, Mannedorf, Switzerland) in triplicate for each sample. Trolox was used as a reference compound, and the results of the ABTS assay were expressed as a µM of Trolox equivalent (TE)/mg of the sample as indicated in [37].

### 2.8. Cell Culture and MTT Test

U937 cells (human leukemia cell line; ATCC, Rockville, MD, USA) were cultured in RPMI 1640 medium (Gibco-BRL, USA, Life Technologies, Segrate Milano, Italy) supplemented with 10% (*v*/*v*) heat-inactivated fetal bovine serum (FBS, Gibco-BRL, USA, Life Technologies, Segrate Milano, Italy), 0.2 M L-glutamine (diluted 100 times), and 1% penicillin–streptomycin (Sigma-Aldrich, Merck Life Science S.r.l., Milano, Italy) at 37 °C in a 5% CO_2_ atmosphere with 90% humidity. These conditions were optimal for U937 cell growth, and cells were split every four days. To simulate monocyte differentiation into macrophages, U937 cells were treated with 5 ng/mL of PMA (Phorbol Myristic Acid, Merck Life Science S.r.l., Milano, Italy) for 72 h, which induced adhesion to the plate, allowing for the transition from circulating cells to adherent cells. Moreover, the cells were subsequently treated with an RPMI 1640 medium containing 0% FBS for 24 h. To test the effect of different concentrations of proteins and hydrolysates on cell vitality after 48 h (i.e., the duplication time of the U937 cell lines), an MTT analysis was carried out according to the supplier’s protocol. The study was performed in duplicate, both with technical triplicates [38].

## 3. Results

### 3.1. SDS-PAGE

**Figure 1:**
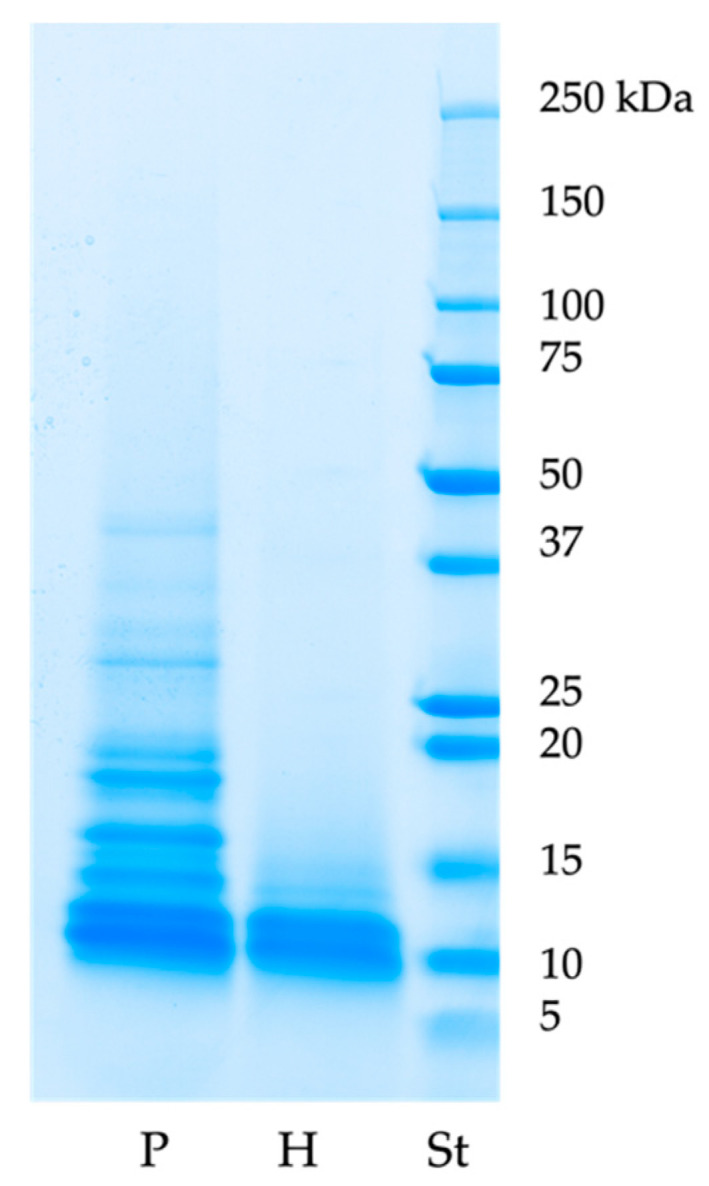
SDS-PAGE profiles of proteins (P), hydrolysates (H), and protein ladder (St).

Protein and hydrolysate profiles obtained through SDS-PAGE are shown in Figure 1. The protein profile shows proteins with molecular weights below 50 kDa. The protein profile shows proteins (P) with molecular weights below 50 kDa. Three bands of weights between 37 and 25 kDa can be seen; of these, only one is well-defined. Between 20 and 15 kDa, three somewhat visible bands are present. In addition, three bands weighing less than 15 kDa are visible; those around 10 kDa, the most visible, are shared with the SDS-PAGE profile of hydrolysates (H) and are probably compatible with smaller fragments of seed storage proteins. From the SDS-PAGE profile of hydrolysate, it is possible to confirm the efficacy of the enzymatic hydrolysis: no bands at higher molecular weight appear.

### 3.2. MALDI-TOF

**Figure 2:**
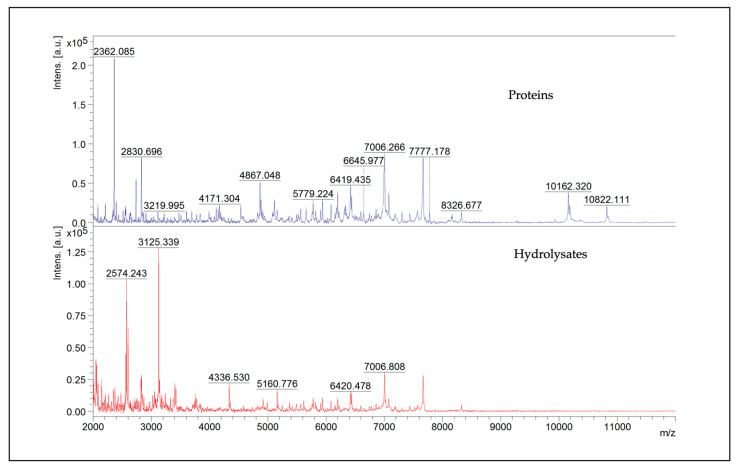
Representative MALDI-TOF MS mass spectra in the *m*/*z* region 2000–20,000 of proteins and hydrolysates; in particular, the image represents a zoom between 2000 and 12,000 *m*/*z*.

Since the SDS-PAGE technique is limited when applied to low-molecular-weight samples, the MALDI-TOF analysis was performed to identify proteins and fragments weighing between 700 Da and 20 kDa. Two methods of analysis were used. For the analysis of the molecular weight range between 700 and 3500 *m*/*z*, the reflectron- positive analysis was applied. The range between 2000 and 20,000 *m*/*z* was studied with the linear positive method. Both methods were used with a laser power of 80% and a laser frequency of 2000 Hz. The mass spectra are the summation of 6000 shots per replicate. Proteins and hydrolysates were analyzed using three technical replicates of each, and two extracts were compared to assess the reproducibility of the sample preparation method. The representative spectra of proteins and hydrolysates obtained through the analysis with the linear positive method are shown in Figure 2.

**Figure 3:**
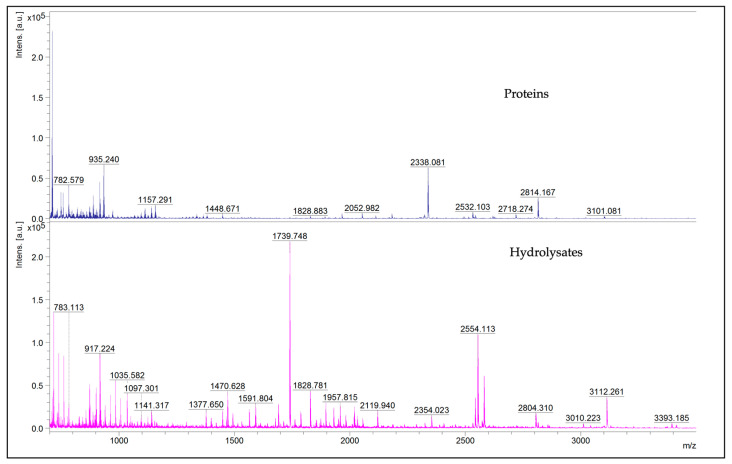
Representative MALDI-TOF MS mass spectra in the *m*/*z* region 700–3500 of proteins and hydrolysates.

From the analysis of the mass lists of the peaks obtained via the MALDI-TOF MS, the absence of peaks higher than 16,000 Da can be highlighted. Figure 3 reports the representative mass spectra of proteins and hydrolysates obtained through reflectron-positive method analysis.

**Figure 4:**
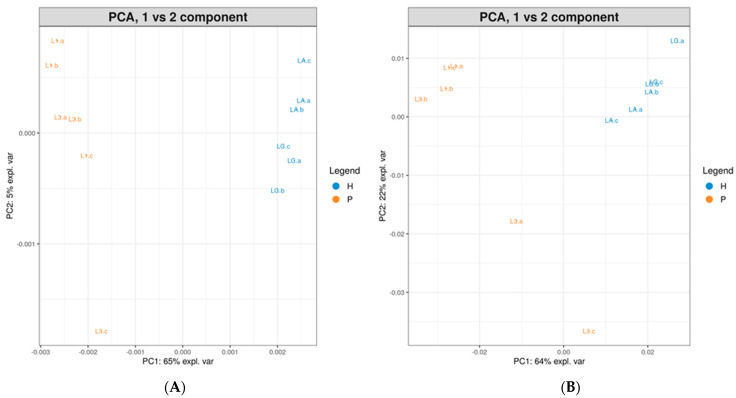
PCA plots (Pc1 vs. PC2) of proteins (P, orange) and hydrolysates (H, blue) analyzed through linear positive method (**A**) and reflectron positive method (**B**). Biological replicates (L1 and L3, LA and LC) are shown in technical triplicate (a, b, and c).

The principal analysis of proteins and hydrolysates spectra was carried out to identify differences or similarities between hydrolysates obtained through enzymatic digestion and low-molecular-weight protein fragments. The box plots (Figure 4) show that proteins’ low-molecular-weight fractions can be distinguished from those obtained after enzymatic hydrolysis.

The top loadings on the first and second principal components (PC1 and PC2) obtained allowed for the identification of the 25 characteristic signals of proteins and hydrolysates (Table 1).

### 3.3. Hydrolysates Identification and Biological Activity Estimation

To identify the hydrolysates generated by pepsin and subsequently analyzed using MALDI-TOF, the proteins that we have previously identified in our recent study [15] and that can be ascribed to the bands observed in the SDS-PAGE (Figure 1) were subjected to in silico digestion. Notably, the following were digested in silico with the web-accessible tool PeptideMass: Edestin 1 (A0A090DLH8), Edestin 2 (A0A090CXP9), Edestin 3 (A0A219D3H6), Sucrose-binding protein-like (XP_030493178.1), Vicilin C72 (XP_030508280.1) and, NADPH-dependent aldehyde reductase 1 chloroplastic-like (XP_030506286.1). Peptides resulting from in silico digestion were matched to the peaks identified with the MALDI-TOF analysis based on their molecular weights. The peptides were investigated to extend the research to other biological activities, as well as the antioxidant activity with the ABTS assay and the in vitro test on U937 cells; the peptides were investigated through BIOPEP. The results of the in silico digestion and BIOPEP research are reported in Table 1. In particular, 27 matches could be identified between the peptides obtained with the MALDI-TOF mass spectrometry and those obtained by in silico digestion; 18 are unique.

**Figure 5:**
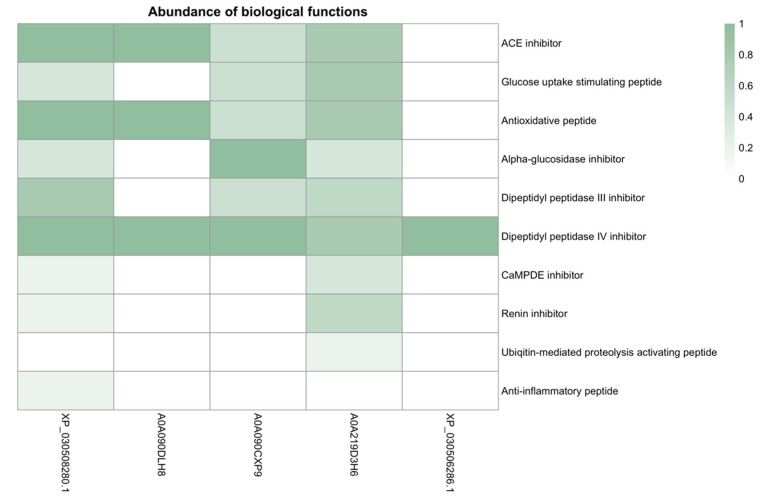
The heatmap represents the relative abundance of different biological activities of peptides obtained from five hemp proteins. Each cell in the map indicates the proportion of biological activity to the number of peptides identified for a given protein. Colors vary in intensity to reflect this proportion, with darker shades indicating higher abundance. Edestin 1 (A0A090DLH8), Edestin 2 (A0A090CXP9), Edestin 3 (A0A219D3H6), Vicilin C72 (XP_030508280.1), and NADPH-dependent aldehyde reductase 1 chloroplastic-like (XP_030506286.1).

Some biological functions identified, such as an Angiotensin-Converting Enzyme inhibition and Dipeptidyl Peptidase IV inhibition, are highly represented in almost all proteins examined. Other activities, such as Dipeptidyl Peptidase III inhibition and ubiquitin-mediated proteolysis activation, show limited distribution (Figure 5).

### 3.4. Antioxidant Activity Estimation Assay

The ABTS assay is based on the ability of a hydrogen donor to reduce the radical form ABTS•+ after proper preparation before the assay. This reaction follows a colorimetric pattern, transitioning from an intensely green ABTS•+ solution to a colorless ABTS solution. An ANOVA was used to prove statistical differences between the proteins and hydrolysates. The reported values represent the mean values of four technical replicates derived from two biological extracts, with the standard deviation. The results of the ABTS assay, expressed as μMTE/mg sample, are reported in Table 2. The results show a statistically significant difference (*p* = 0.0262, N = 4) between the samples: hydrolysates have higher antioxidant activity than proteins.

### 3.5. MTT Test and Morphological Representation

**Figure 6:**
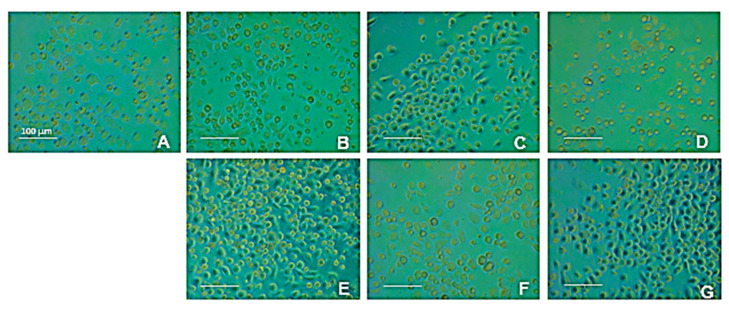
The figure shows a typical experiment. U937 cells are treated with different concentrations of the stimuli, proteins (**B**–**D**) and hydrolysates (**E**–**G**). Image (**A**) represents untreated control. The cells were treated with proteins (**B**–**D**), 0.1, 0.01, and 0.001 mg/mL, respectively, and with hydrolysates (**E**–**G**), 0.1, 0.01, and 0.001 mg/mL, respectively (magnification 20×, scale bar = 100 μm).

The MTT test (3-(4,5-dimethylthiazol-2-yl)-2,5-diphenyltetrazolium bromide test) is an assay usually used to measure the metabolic activity of cells or rather to estimate their viability and, consequently, the cytotoxicity of specific compounds. The assay is based on the cell’s ability to convert the yellow MTT dye into a purple formazan product. The intensity of the purple, measured as absorbance, is proportional to the number of viable cells. The results of the MTT test are reported in Table 3 and show a significant decrease in cell viability, *p* < 0.1, for treatment with proteins at the concentration of 0.1 mg/mL and *p* < 0.01 for treatment with protein concentration 0.01 mg/mL and 0.001 mg/mL. On the contrary, hydrolysates do not affect cell viability. Figure 6 shows the morphology of the cells treated and untreated with the compounds.

## 4. Discussion

Hempseeds are noteworthy as a source of nutrients: they provide a useful source of proteins, carbohydrates, lipids, fibers, and micronutrients. Moreover, these seeds elicit beneficial biological effects in humans upon the digestion of their constituents.

In this research, we focused on water-soluble proteins and their hydrolysates to confirm the biological activity of such nutrients using a method that can partially simulate what happens during human digestion. Protein separation by SDS-PAGE is one of the screening analyses for proteins. The band, observable in the SDS-PAGE profile of the water-soluble protein fraction, between 50 kDa and 37 kDa, could be ascribed to fragments of Edestin 1, Vicilin C72-like, and sucrose-binding proteins, as already described in a recent work [4]. The bands weighing between 37 kDa and 25 kDa can be attributed to fragments of the three isoforms of Edestin, Vicilin C72-like, and sucrose-binding proteins. Moreover, in the same recent work, in correspondence to bands of the same molecular weights, we were able to identify also NADPH-dependent aldehyde reductase 1 chloroplast-like and Aspartic proteinase A1-like proteins of *Cannabis sativa*. Interestingly, the most intense bands are visible at molecular weights below 20 kDa. These bands have also been found in [28]; here, they were visualized with a low optical density in the water-soluble fraction, contrary to very intense in the basic solution–soluble fraction (0.1 M NaOH, pH 11–11.5). Finally, a similar smear area of around 10 kDa can be seen for both protein and hydrolysate profiles. Proteins and hydrolysates were analyzed with MALDI-TOF to further investigate the low-molecular-weight proteins that were not well resolved in SDS-PAGE. In contrast to what is visible in the SDS-PAGE gel image of proteins, no peaks over 16,753 Da are present in the mass list obtained via MALDI-TOF. In addition, only a few isolated peaks around 13,000, 11,000, and 10,000 Da are observable. Below 10,000 Da, there are many peaks at different intensities.

After processing the *m*/*z* and peak intensity data from both methods using the GeenaR software (https://proteomics.hsanmartino.it/geenar/), the samples are separated in both plots. Analyzing samples with the linear positive method and considering all the peaks in the mass range from 2000 Da to 50,000 Da, it is possible to obtain a total PCA variance of 70%. Regarding mass range from 700 Da to 3500 obtained with the reflectron-positive method, a total PCA variance of 86% is possible. The principal component analysis, in particular, was obtained by considering the intensity of the *m*/*z* peaks and their membership in the protein or hydrolysate group, resulting in the top 25 loadings. This further analysis made it possible to discriminate peptides obtained via hydrolysis from those already present as low-molecular-weight fragments in the protein fraction. From the study of the MALDI-TOF mass spectra of hydrolysates, it is possible to identify peaks 1141.344 as a fragment of the Beta Subunit of RNA Polymerase *Cannabis sativa* (A0A0C5ARQ8) and 1470.627 as a fragment of Acyl-Activating Enzyme 5 *Cannabis sativa* (H9A1V7) [11]. Moreover, after in silico digestion, it was possible to identify abundant fragments of different hempseed storage proteins. Those fragments can exert different biological activities: the main suggested activities are the inhibition of DPP-IV, DPP-III, and ACE; antioxidant activity; and finally, glucose-uptake-stimulating activity [22,24,27]. The ABTS assay has also confirmed the antioxidant activity of both proteins and hydrolysates. The data indicate that hydrolysates have higher antioxidant activity than proteins, consistent with findings in the literature [1,31].

Moreover, hempseed proteins and their hydrolysates proved the absence of pro-inflammatory effects when interacting with the immune system, as simulated through the U937 cell line model. These findings are particularly relevant to autoimmune diseases, where dysregulated immune responses, often involving macrophages, play a key role. The U937 cell line—widely used as an in vitro model for monocyte and macrophage biology—has proven to be instrumental in understanding macrophage function in autoimmune conditions, such as systemic lupus erythematosus (SLE), rheumatoid arthritis (RA), and inflammatory bowel diseases (IBDs) [20,21]. In IBDs, such as Crohn’s disease and ulcerative colitis, macrophages are central to the chronic inflammation observed in the gastrointestinal (GI) tract. Here, U937 cells provide a robust platform to evaluate how dietary components, such as hempseed protein hydrolysates, can modulate macrophage activity and inflammatory signaling pathways in the gut [26].

The immunomodulatory potential of hempseed protein hydrolysates was supported by the observation that hydrolysates showed higher antioxidant activity than undigested proteins, as shown through ABTS assays. These hydrolysates also lacked inflammatory effects on U937 cells, indicating their potential for therapeutic applications in managing inflammation-associated autoimmune diseases.

Dietary proteins and peptides can play a dual role in gastrointestinal autoimmune diseases, such as celiac disease. While specific peptides, such as those from gluten, trigger immune responses, bioactive peptides, like those derived from hempseed proteins, may offer protective and modulatory effects. By using the U937 cell line as a model for gut-associated immune interactions, this study supports the idea that hempseed-derived peptides can interact with immune cells without activating pro-inflammatory responses [25].

These insights suggest promising implications for clinical applications. Hempseed proteins and their hydrolysates show anti-inflammatory and antioxidant properties that are particularly significant in autoimmune diseases characterized by chronic inflammation. The interaction of these bioactive peptides with the immune system in the gut highlights their potential in managing diseases such as IBDs, where the preservation of immune homeostasis is critical.

## 5. Conclusions

This study validates the biological activity of hempseed protein hydrolysates, particularly their antioxidant properties and lack of inflammatory effects on macrophage-like U937 cells. These results underscore the potential role of hempseed-derived peptides in dietary strategies to mitigate inflammation in autoimmune diseases. By integrating the findings with the role of U937 cells as a model for studying immune responses, this research highlights the importance of dietary interventions in modulating immune function in conditions like IBDs and celiac disease. Further investigations are warranted to explore the translational applications of these peptides in clinical settings, particularly their potential to contribute to managing chronic inflammatory and autoimmune conditions affecting the GI tract.

## Figures and Tables

**Table 1:** Mass signals from proteins and hydrolysates samples, analyzed using linear positive and reflectron methods, corresponding to the top 25 loading on PC1.

Samples	Linear Positive	Reflectron Positive
Proteins	2362.6657 2830.4573 2737.0570 4867.8664 10,163.7268 4864.3402	717.1909 1740.7847 761.1945 1739.7938 739.1931 985.2238 1741.7972
Hydrolysates	3125.3030 2574.2319 2572.8877 3123.2903 2599.9435 2602.2150 2377.1769 4336.9857 2148.5979 2563.6556 2348.9317 3023.9891 3403.9005 2261.3035 2085.7081 2049.6929 2476.9148 2312.2604 2410.8697	711.1693 709.1610 712.1759 935.2570 713.1834 933.2470 2339.1257 782.6017 710.1659 2338.1290 936.2611 758.5947 2340.1256 934.2536 1157.3256 889.2501 749.1247 1159.3248

**Table 2:** Antioxidant activity of proteins and hydrolysates samples expressed as μMTE/mg sample, determined via ABTS assay, with standard deviation (*p* = 0.0262, N = 4).

	μMTE/mg
Proteins	81.04 ± 10.63
Hydrolysates	97.95 ± 4.48

**Table 3:** Effects of proteins (A) and hydrolysates (B) on the U937 cell line (concentration range from 0 mg/mL, control, to 0.001 mg/mL). Results are reported as absorbance ± standard deviation. Statistical significance was calculated vs. untreated control (0 mg/mL).

Treatment Concentration (mg/mL)	Proteins Absorbance (N = 6)	Hydrolysates Absorbances (N = 5)
0	0.361 *±* 0.025	0.361 ± 0.025
0.1	0.336 ± 0.023 (n.s. *p* = 0.067)	0.343 ± 0.025 (n.s. *p* = 0.2)
0.01	0.323 ± 0.007 (*p* = 0.0028)	0.364 ± 0.025 (n.s. *p* = 0.8)
0.001	0.283 ± 0.025 (*p* = 0.00003)	0.335 ± 0.023 (n.s. *p* = 0.07)

## Data Availability

The data can be made available upon reasonable request.
